# Biomarkers and algorithms for diagnosis of ovarian cancer: CA125, HE4, RMI and ROMA, a review

**DOI:** 10.1186/s13048-019-0503-7

**Published:** 2019-03-27

**Authors:** Vincent Dochez, Hélène Caillon, Edouard Vaucel, Jérôme Dimet, Norbert Winer, Guillaume Ducarme

**Affiliations:** 1 Service de Gynécologie-Obstétrique, CHU de Nantes, 44093 Nantes, France; 2 Service de Biochimie, CHU de Nantes, Nantes, France; 3 Centre de recherche clinique, Centre Hospitalier Départemental Vendée, La Roche sur Yon, France; 4 Service de Gynécologie-Obstétrique, Centre Hospitalier Départemental Vendée, La Roche sur Yon, France

**Keywords:** HE4, CA125, ROMA, RMI, Ovarian cancer

## Abstract

Ovarian cancer is the 5th leading cause of death for women with cancer worldwide. In more than 70% of cases, it is only diagnosed at an advanced stage. Our study aims to give an update on the biological markers for diagnosing ovarian cancer, specifically HE4, CA 125, RMI and ROMA algorithms.

Serum CA125 assay has low sensitivity in the early stages and can be increased in certain conditions such as menstruation or endometriosis. The level of HE4 is overexpressed in ovarian tumors. Its specificity is 94% and its level is not affected by endometriosis cysts. The combined measures of CA125 and HE4 have proved to be highly efficient with an area under the curve (AUC) of up to 0.96. Furthermore, this combined measure of CA125 can correct the variations in HE4 which are due to smoking or contraception combining estrogen plus progestin. While the specificity of RMI sometimes reaches 92%, the rather low AUC of 0.86 does not make it the best diagnostic tool. The specificity of ROMA is lower than HE4 (84% compared to 94%).

To date, the most efficient biological diagnostic tool to diagnose ovarian cancer is the combination of CA125 and HE4.

## Introduction

Ovarian cancer is the 5th worldwide leading cause of death of women due to cancer [1]. In more than 70% of cases, it is diagnosed at an advanced phase. The prognosis for ovarian cancer remains poor overall, with a 46% 5-year survival rate [2]. The prognosis is closely related to the stage at diagnosis: survival rate of > 70% after 5 years for stage I or II, survival rates between 20 and 40% for stage III or IV [3, 4].

More than 90% of benign tumors are found in premenopausal patients who have been operated on, whereas in postmenopausal patients only 60% of tumors are benign [5]. It seems essential to differentiate early malignant ovarian tumors to benign ovarian tumors. Exams are therefore needed which must be prioritized in order to advise the patient (monitoring, treatment or survey) according to the lesion, and first and foremost to the clinical history of the patient [6].

Concerning laboratory exams, several tumor biomarkers have been evaluated. The Carbohydrate Antigen 125 (CA125) was first described in the early 1980’s [7]. In cases of ovarian cancer, serum CA125 level may be elevated, but this marker has a low sensitivity in the early stages of ovarian cancer [8]. Increased CA125 levels are also reported in other physiological or pathological conditions, such as menstruation, pregnancy, endometriosis and inflammatory diseases of the peritoneum [9]. Other biomarkers have been developed in order to improve specificity for ovarian carcinomas, such as the Human Epididymis Protein 4 (HE4) [10]. This biomarker is reported to be overexpressed in ovarian cancer [11]. Although the specificity of these markers is rather reliable, they are not very sensitive. For this reason, algorithms, RMI (Risk of Malignancy Index) and ROMA (Risk of Ovarian Malignancy Algorithm), were developed in an attempt to improve the inherent characteristics of these biomarkers.

HE4 was found to be a reliable biological marker for detecting ovarian cancer (level of evidence [LE]1) and ROMA algorithm was more sensitive but less specific than HE4 alone (LE2) for the French National College of Obstetricians and Gynaecologists (CNGOF) [12]. CNGOF also concluded that complementary studies should be necessary before using HE4 on a routine basis (Grade B). Our study intends to provide a comprehensive update for ovarian cancer diagnosis using biomarkers (HE4 and CA125) and algorithms (RMI and ROMA). The sensitivity, specificity, positive and negative predictive values of these tools have been collated in Table 1 for CA125 and HE4 and Table 2 for RMI and ROMA algorithms. Only the values of some cited articles are referenced in order to lighten the table and make it readable.

**Table 1:** Diagnostic performance of CA125, HE4 and combination of CA125 + HE4 in the subset of studies cited in this article

	Systematic review or meta-analysis	CA125					HE4					CA125 + HE4				
		Se (%) (95% IC)	Sp (%) (95% IC)	PPV (%)	NPV (%)	AUC (95% IC)	Se (%) (95% IC)	Sp (%) (95% IC)	PPV (%)	NPV (%)	AUC (95% IC)	Se (%) (95% IC)	Sp (%) (95% IC)	PPV (%)	NPV (%)	AUC (95% IC)
Ferraro et al. [14]	X	79 (77–82)	78 (76–80)				79 (76–81)	93 (92–94)				82 (78–86)	76 (72–80)			
Dikmen et al. [15]		63				0.78	78				0.93					
Chen et al. [19]		93	67			0.93 (0.88–.97)	73	99			0.96 (0.93–1)	97	66			0.96 (0.93–1)
Yanaranop et al. [31]		84	53	41	89	0.81 (0.74–0.87)	66	86	65	87	0.82 (0.76–.89)					
Wilailak et al. [32]						0.87 (0.80–0.93)					0.89 (0.84–0.95)					0.89 (0.84–0.95)
Wang et al. [36]	X	79 (74–84)	82 (77–87)			0.87 (0.84–0.90)	76 (72–80)	94 (90–96)			0.89 (0.86–0.92)					
Zhen et al. [37]	X	74 (72–76)	83 (81–84)			0.85	74 (72–76)	90 (89–91)			0.89					
Abdel-Azeez et al. [45]		73 (57–86)	79 (58–93)			0.90 (0.82–0.97)	83 (68–93)	88 (68–97)			0.95 (0.90–1)	90	79			
Holcomb et al. [46]		85 (69–95)	59 (52–66)	27	96		65 (46–80)	92 (87–95)	58	94		91	55	26	97	
Moore et al. [48]			61			0.84 (0.77–0.90)		78			0.91 (0.86–0.95)		81			0.91 (0.87–0.96)
Goff et al. [52]		79 (67–88)	76 (68–83)	63	87		58 (45–70)	94 (88–97)	83	81						
Meys et al. [55]	X															
Van Gorp et al. [56]		80 (72–85)	82 (76–86)				75 (67–81)	83 (78–88)								
Al Musalhi et al. [57]		79	62	38	91	0.81	71	90	68	91	0.82					
Moore et al. [62]												89 (85–93)	75 (70–79)	60	94	
Li et al. [64]	X	77 (58–89)	84 (76–90)			0.88 (0.85–0.91)	79 (74–84)	93 (87–96)			0.82 (0.78–0.85)					
Wei et al. [66]		85	92	91	89		75	98	96	85						
Sandri et al. [67]		91	71			0.90 (0.86–0.93)	83	91			0.92 (0.89–.95)					

*CA125* carbohydrate antigen 125, *HE4* human epididymis protein 4, *Se* sensitivity, *Sp* specificity, *PPV* positive predictive value, *NPV* negative predictive value, *AUC* area under the curve

**Table 2:** Diagnostic performance of RMI and ROMA algorithms in the subset of studies cited in this article

	Systematic review or meta-analysis	RMI					ROMA				
		Se (%) (95% IC)	Sp (%) (95% IC)	PPV (%)	NPV (%)	AUC (95% IC)	Se (%) (95% IC)	Sp (%) (95% IC)	PPV (%)	NPV (%)	AUC (95% IC)
Ferraro et al. [14]	X										
Dikmen et al. [15]							88				0.96
Chen et al. [19]							97	80			0.97 (0.95–1)
Yanaranop et al. [31]		78	80	60	90	0.88 (0.83–0.93)	84	69	52	91	0.86 (0.81–0.91)
Wilailak et al. [32]						0.84 (0.77–0.91)					0.86 (0.81–0.91)
Wang et al. [36]	X						85 (81–89)	82 (77–87)			0.91 (0.88–0.93)
Zhen et al. [37]	X										
Abdel-Azeez et al. [45]											
Holcomb et al. [46]											
Moore et al. [48]											
Goff et al. [52]											
Meys et al. [55]	X	75 (72–79)	92 (88–94)								
Van Gorp et al. [56]											
Al Musalhi et al. [57]		77	82	56	93	0.85	75	88	65	92	0.84
Moore et al. [62]											
Li et al. [64]	X						89 (84–93)	83 (77–88)			0.93 (0.90–0.95)
Wei et al. [66]							94	93	90	86	
Sandri et al. [67]							89	81			0.93 (0.90–0.96)

*RMI* risk of malignancy index, *ROMA* risk of ovarian malignancy algorithm, *Se* sensitivity, *Sp* specificity, *PPV* positive predictive value, *NPV* negative predictive value, *AUC* area under the curve

## Discussion

### Carbohydrate antigen 125 (CA 125)

Carbohydrate Antigen 125 (CA 125), sometimes named as Cancer Antigen 125 or Tumor Antigen 125, is a mucin-type glycoprotein, produced by the MUC16 gene, and associated with the cellular membrane.

This biomarker is most often used for ovarian lesions. It has been used in the early 1980’s when Bast et al. [7] specifically isolated the monoclonal antibody OC125 in cancerous ovarian tissue compared to healthy ovarian tissue. Its upper limit is 35 U/mL in pre and post-menopausal patients [13]. However, this measurement is not very sensitive in the early phases of ovarian cancer (only reported to be elevated in 23 to 50% of stage I cases) [10]. In addition, elevated serum CA125 levels may be observed in other physiological or pathological conditions (menstruation, pregnancy, endometriosis, inflammatory diseases of the peritoneum) [9]. In a meta-analysis by Ferraro et al. [14], the specificity of CA125 for detecting ovarian cancer was 78% (95%CI 76–80). To describe tumor markers and screening tests, the Receiver Operating Characteristic (ROC) area under the curve (AUC) is frequently employed since it represents a useful graphic tool for comparing biomarkers and algorithms. The ROC measures the discrimination of a test, i.e. its ability to distinguish between having disease and not having disease for a given patient. In the study by Dikmen et al. [15], the AUC for CA125 was rather weak (0.78), suggesting that it was probably not the ideal marker for diagnosing ovarian cancer.

Serum CA125 levels were frequently measured when ovarian cysts are observed, in order to rule out a malignant tumor. But for the past several decades, elevated serum CA125 levels have been seen in endometrioma, thus giving a high rate a false positives [16]. This was confirmed in a recent Cochrane review which reported that among the 97 biomarkers studied, CA125 was the only marker which is elevated in cases of endometrioma with 40% sensitivity and 91% specificity with a cut off limit of 35 U/mL [17]. In another very recent meta-analysis, Hirsch et al. [18] demonstrated that CA125 should be useful to diagnose endometriosis, especially with an increasing sensitivity corresponding simultaneously with the disease phase. Chen et al. [19] reported that CA125 levels were significantly higher in the group with endometriotic cysts compared to group with other benign ovarian tumors (49.7 U/mL vs. 21.6 U/mL).

Since CA125 has been a tumor marker for several decades, the changes in its levels according to the patient’s demographic characteristics and lifestyles have been evaluated numerous times [20, 21]. Smoking does not appear to modify serum CA125 levels [22, 23]. Whereas CA125 may vary during menstrual cycles, it has been demonstrated that levels are not affected by a contraception combining estrogen plus progestin [21, 24]. Likewise, the body mass index does not appear to modify CA125 levels [25].

In practice, CA125 is often measured in cases of ovarian cysts, but according to its low specificity and the observed increased levels in different physiological situations, it is not considered as a very good differentiating biomarker for ovarian tumors. For this reason, new biomarkers have been evaluated in an attempt to improve early diagnosis of ovarian cancer [26].

### Human epididymis protein 4 (HE4)

Human Epididymis Protein 4 (HE4) is a new biomarker which has been currently evaluated for diagnosing ovarian malignant tumors [10]. It is a glycoprotein belonging to the family of whey acidic four-disulfide core proteins, accounting for its alternative name of WFDC2 and the larger protein family called “WAP” for whey acidic proteins. The main genes coding for the WAP proteins are mainly located on chromosome 20q12–13.1 [27]. Present in whey, these proteins are called WAP, which is composed of around 50 amino acids, and its biological function has not yet been completely identified [28].

HE4, which contains 2 WAP domains, was initially isolated in the epididymis and might play a role in sperm maturation [29]. This biomarker is weakly expressed in the epithelium tissues of respiratory and reproductive organs, but is overexpressed in ovarian tumors, especially in endometrioid ovarian cancer [11]. In addition, it appears that HE4 is not as strongly expressed in clear cell ovarian carcinomas as in other epithelial ovarian cancers [30]. Yanaranop et al. [31] reported a specificity of 86% for HE4, and the AUC was higher than CA125 alone, with values of 0.893 and 0.865, respectively [32]. These data, in accordance with those reported in a recent Italian multicentre study included 387 patients, showed that HE4 for diagnosing ovarian epithelial cancer appeared more reliable than CA125 [33].

To measure this biomarker, an immune-enzymatic (EIA) assay described by the Fujirebio lab was developed [34]. Other immunological methods have since been developed and evaluated such as electrochemiluminescent (ECLIA) or chemiluminescent microparticle immunoassay (CMIA). A recent study evaluated these different measurement techniques for HE4 levels [35]. The authors reported that ECLIA and CMIA were well compared with the reference method and could be routinely used in practice. However, significantly different mean values were reported according to the immunological assay used; HE4 marker levels might always be interpreted according to the immunological assay employed. The cut off level of 70 pmol/L is often used for pre-menopause patients and 140 pmol/L for menopause patients; but sometimes the threshold level of 140 pmol/L is employed, or even other close but outlying values. The threshold value of HE4 used, including whether or not the menopausal status, is left to the choice of clinicians [36, 37]. Indeed, these values can be used whatever the immunological method performed. Nevertheless, given the fact that is proven that HE4 increases significantly over the age, the use of 2 thresholds (70 and 140 pmol/l) seems preferable than the use of a single threshold (140 pmol/l) [38, 39]. In conclusion, the review of the literature demonstrates that all meta-analyses always take into account the different methods used to measure HE4 and the results must be carefully handled.

Although CA125 levels seem to be elevated in endometrioma, HE4 levels appear to remain stable [19, 40, 41]. HE4 levels in patients with endometrioma were comparable to levels in patients with other benign ovarian cysts (53.0 pmol/L vs. 52.8 pmol/L) [19], quite understandable since the gene coding for HE4 is not overexpressed in endometriotic lesions [42]. A recent study has also confirmed that serum HE4 was a better diagnostic biomarker than CA125 in ovarian cancer patients with endometriosis [38].

Variations of HE4 levels in variable situations were also evaluated. Whereas Bolstad et al. [38] reported modified levels of HE4 according to Body Mass Index (BMI), Ferraro et al. [25] did not report significant different levels of HE4 in 103 patients according to BMI, probably explained by inclusion of men and women in this study which should constitute a bias. In conclusion, serum HE4 levels appeared to not be modified by the BMI, as CA125.

Nevertheless, in opposite to CA125, smoking seems to be a significant factor which affects serum HE4 variations [43]. HE4 level is increased from 20 to 30% in smokers compared to non-smokers [23, 38, 44]. So, HE4 level should be always interpreted carefully in smokers, since it could be misinterpreted as a false positive result.

In opposite to CA125, contraceptive use contributes to variations in HE4 levels. Ferraro et al. [24] reported a significantly lower level of HE4 in patients using oral contraception compared to patients using other contraceptive methods (*p* = 0.008). Therefore, in order to misinterpret HE4 levels, it appears noteworthy to include the contraceptive method in the patient’s clinical history.

### Associating HE4 + CA125

Three meta-analyzes or systematic reviews show the use of HE4 or CA125, always with different thresholds for HE4 [14, 36, 37]. The combined use of these markers is only sometimes studied [45–47]. Chen et al. [19] reported a specificity of 65.7% using the ECLIA immunological method with a cut-off value for HE4 of 140 pmol/L. In a different study using another technique to assess serum HE4, the specificity of the association CA125 and HE4 was much better (80%) [48]. However, the ROC AUC when combining the two markers was high, varying from 0.96 (IC95% 0.93–1) [19] to 0.91 (IC95% 86.7–96.0) [48]. Thus, the association of CA125 and HE4 is a useful diagnostic tool in ovarian cancer and may be used in addition to each biomarker.

These results are confirmed by another study analyzing each marker separately or in combination [49]. That is, in case of increased HE4 and CA125, the specificity was better. The use of a third marker, for example clinical, could also improve the detection of these ovarian cancers.

Besides its use diagnosing ovarian cancer, the measurements of the association HE4 + CA125 can also be useful in differential diagnosis of different ovarian tumors. Anastasi et al. [50] studied 57 patients with endometrioma; all had increased CA125 levels, while the HE4 levels remained normal. Another very recent study confirmed the significant difference between HE4 and CA125 levels in endometrioma cases [51]. Thus, endometrioma could be suspected whenever CA125 levels are elevated while HE4 levels remain normal. In addition, while HE4 level varies in smokers and in contraceptive combining estrogen plus progestin users, simultaneous CA125 evaluation which is not affected by these variables should allow better interpretation of abnormal HE4 levels.

In conclusion, it seems worthwhile to measure both markers in cases of suspected benign ovarian tumors: an increased value of the 2 markers being suggestive of an ovarian cancer As suggested in a recent study by Goff et al., thresholds of 70 and 140 pmol/l according to menopausal status and 35UI/ml for CA125 seems preferable [52]. Furthermore, the use of this combined HE4 and CA125 assay may also be of major interest in ovarian cancer screening in the general population, as shown by Urban et al. [44]. However, in this context, the place of transvaginal ultrasound remains to be clarified, and the use of a second positive test could reduce sensitivity (key principle of a screening test).

### Risk of malignancy index (RMI)

RMI was proposed in 1990 by Jacobs et al. [53], using CA125, ultrasound findings and menopausal status according to the formula: RMI = U x M x CA125 with U = ultrasound score (U = 0 if ultrasound score = 0, U = 1 if ultrasound score = 1, U = 3 if ultrasound score 2 to5), M = menopause status (M = 1 for pre-menopausal women, M = 3 for post-menopausal women). A RMI score above 200 proved to have a strong association with a high risk of malignancy (sensitivity 85.4% and specificity 96.9%).

Another study performed in 2012 on nearly 1000 patients demonstrated that the ultrasound assessment was superior according to IOTA (International Ovarian Tumor Analysis) criteria compared to RMI [54]. These data were confirmed in a recent meta-analysis published in 2016 [55]. This study of almost 20,000 ovarian tumors reported better results with the use of ultrasound criteria (sensitivity 93% and specificity 80%) compared to that obtained with the RMI algorithm (sensitivity 75% and specificity 92%). The specificity of RMI for diagnosing ovarian cancer is rather high, 92.4% [56] and 92% in a recent meta-analysis [55]. This specificity could be increased by modifying the threshold level for malignancy (using 250, as reported by Al Musalhi et al. [57]).

Since 1990 with Jacobs’ suggestion to use RMI algorithms, several variations in RMI formula have been developed. Chopra et al. [58] studied 100 patients, using a modified RMI with the maximum values for the U et M parameters were 4 instead of 3, which increased the sensitivity to 96.7% with 84% specificity, and a positive predictive value of 85.5%. Recently, the use of four different RMI formulas showed the same results for sensitivity. The positive likelihood ratio was reported between 3.52 and 4.41 [59].

For screening tests, the use of ROC AUC seems to be a useful indicator for detecting cancer. That of RMI remains weak (0.86) [60] compared to other biomarkers which emphasize that RMI algorithm don’t appear to be the most useful diagnostic tool for ovarian cancer.

According to this hypothesis, a 2-steps triage model, associating ultrasound findings first, RMI could improve screening results. Using this 2-step model, a recent study in 2016 reported an improved detection rate of ovarian cancer through 72 to 85% [61].

Finally, concerning this RMI algorithm or modified RMI algorithms, the variable U may be equal to 0, the RMI score may be 0 (RMI = U x M x CA125). This value 0 may sometimes seem aberrant in the interpretation of a score. Nevertheless, the RMI score is based on a threshold (200). The calculation of specificity, sensitivity and positive and negative predictive values is not based on the value of the score but on its value below or above that threshold. The value 0 therefore impacts the interpretation of this algorithm only very moderately.

### Risk of ovarian malignancy algorithm (ROMA)

In 2009, Moore proposed a new algorithm: Risk of Ovarian Malignancy Algorithm (ROMA) [62]. He associated HE4 and CA125 levels according to the menopausal status, defined by lack of menstruation or clinical signs of menopause for 6 months.

$$ Pre- menopausal\ Predictive\ Index\left(\mathrm{PI}\right)=-12.0+2.38\times \mathrm{LN}\ \left(\mathrm{HE}4\right)+0.0626\times \mathrm{LN}\left(\mathrm{CA}125\right) $$

$$ Post- menopausal\ Predictive\ Index\left(\mathrm{PI}\right)=-8.09+1.04\times \mathrm{LN}\left(\mathrm{HE}4\right)+0.732\times \mathrm{LN}\left(\mathrm{CA}125\right) $$

$$ Predicted\ Probability\left(\mathrm{PP}\right)=\exp \left(\mathrm{PI}\right)/\left[1+\exp \left(\mathrm{PI}\right)\right]\times 100 $$

Therefore, the ROMA score corresponds to Predicted Probability [PP] and is expressed by a percentage rate. Different cut off levels are proposed for non-menopausal women, and another for women having reached menopause. According to the immunological assay for measuring CA125 and HE4, the cut off levels can differ to classify patients into either a low or an high risk group [63]. In fact, with the Roche Diagnostics Laboratory’s ECLIA method, the cut off level to classify patients in a high risk group was 11.4% for pre-menopausal patients, and 29.9% for menopause patients. Whereas with Abbott Diagnostics Laboratory’s CMIA method, the cut off levels were respectively 7.4 and 25.3% [32]. It is thus indispensable to know which method is used or to refer to the normal values given by the lab performing the tests in order to correctly interpret the results of this algorithm.

In a meta-analysis, the ROMA algorithm was reported with less specificity than that associated with HE4 levels (84% vs. 94%), but a better correlation than with CA125 levels (84% vs. 78%) [64]. In this meta-analysis, the AUC for the ROMA algorithm was better than HE4 or CA125 (0.93, 0.82 and 0.88; respectively), as in the meta-analysis performed by Wang et al. (0.91, 0.89 and 0.87; respectively) [36].

Another meta-analysis by Kaijser et al. [65] demonstrated a ROMA sensitivity between 76 and 86%, while specificity was evaluated between 74 and 95%, in spite of using different methods for measuring the markers. Recently, ROMA was reported having a non-significant different specificity to CA125 (approximately 92.5%) but with significantly improved sensitivity levels: 93.7% vs. 85.0% [66]. For Chen et al. [19], ROMA was more sensitive than HE4, respectively 96.7 and 73.3%, but with less specificity (80% vs. 98.6%). In this same study, AUC for ROMA and HE4 were not significantly different (0.97 and 0.96, respectively). Several more recent studies confirm these results [32, 67]. Nevertheless, it should be noted that it is difficult to interpret these meta-analyses results. When studies have used different measuring techniques for CA125 and HE4 [14, 36, 64], the results thus indirectly implied different ROMA calculations. Therefore, one must be cautious in interpreting cut off levels.

Although the dual measurement of CA125 and HE4 is apparently the best diagnostic tool over and above ROMA algorithms; the fact of including ages in the ROMA model could be a valuable contribution for diagnosing ovarian cancer [68]. It was reported that serum HE4 levels regularly increased with age, without any sudden peak at menopause [38, 39]. Using age in the algorithm, it should be a valuable contribution for evaluating serum HE4 levels.

### Modified risk of ovarian malignancy algorithm: CPH-I and ROMA P

In 2015, the Copenhagen Index (CPH-I) was reported as a novel diagnostic score index in ovarian tumors [69]. It used the same mathematical method than the ROMA algorithm with a Predicted Probability called PP. The CPH-I formula is: CPH-I = − 14.0647 + 1.0649 x log2(HE4) + 0.6050 x log2(CA125) + 0.2672 x age / 10 with PP = e(CPH-I) / (1 + e(CPH-I)).

The ROC AUC were comparable according to the different respective algorithms: CPH-I, ROMA and RMI (0.96, 0.95 and 0.96, respectively). Thus, the introduction of the age in the algorithm did not improve the diagnostic of ovarian cancer. More recently, Chudecka-Glaz et al. [70] evaluated another modified ROMA algorithm, called ROMA P, which took into account the patient’s age and not her menopausal status, according to the formula: ROMA P = exp.(PI) / (1-exp(PI)) × 100 with the predictive index formula PI: PI = A + W(HE4) x ln(HE4) + W(CA125) x ln(CA125), and A, W(HE4) et W(CA125) were the varying coefficients for each decade in function of the patient’s age. ROMA P algorithm had a higher specificity and a higher positive predictive value, but the sensitivity and the negative predictive value were lower than the non-modified ROMA algorithm. Otherwise, the ROC AUC for these two algorithms were comparable (0.923 for the ROMA P vs. 0.934 for ROMA).

## Conclusions

The best biological diagnostic tool today seems to be a combination of CA125 and HE4 levels in order to predict the risk of ovarian cancer in patients with suspected benign ovarian tumors. If the level of CA125 is increased as well as that of HE4, it is necessary to evoke a malignant lesion and therefore to envisage a surgical treatment for an anatomopathological examination. On the other hand, if one of the markers was above the cut-off as long as the other was below the cut-off specified, a simple ultrasound or biological monitoring may be considered. As the HE4 levels increase with advancing age, it might be interesting to establish algorithms which take into account the patients’ age and not her menopausal status. The previously published algorithms (CHP-I or ROMA P) have not proved to be valuable compared to RMI or ROMA algorithms. Serum HE4 levels vary in smokers and in hormonal contraceptive users, thus it seems relevant that this information should always be included in the patient’s clinical history. Nonetheless, since CA125 levels are independent from these variables, the simultaneous measure of these two markers allows the correction of any possible variations in such specific cases.

## Abbreviations

AUC: Area under the Curve
BMI: Body Mass Index
CA125: Carbohydrate Antigen 125
CMIA: Chemiluminescent microparticle immunoassay
ECLIA: Electrochemiluminescent immunoassay
EIA: Immune-Enzymatic assay
HE4: Human Epididymis Protein 4
IOTA: International Ovarian Tumor Analysis
RMI: Risk of Malignancy Index
ROC: Receiver Operating Characteristic
ROMA: Risk of Ovarian Malignancy Algorithm
